# Impact of food safety supervision efficiency on preventing and controlling mass public crisis

**DOI:** 10.3389/fpubh.2022.1052273

**Published:** 2022-12-05

**Authors:** Jian Ding, Ping Qiao, Jiaxing Wang, Hongyan Huang

**Affiliations:** ^1^Faculty of Business and Economics, University of Malaya, Kuala Lumpur, Malaysia; ^2^School of Industrial and Information Engineering, Politecnico di Milano, Milan, Italy; ^3^School of Accounting, Zhongnan University of Economics and Law, Wuhan, China

**Keywords:** food safety, regulatory efficiency, cluster public crises, mediating effects, moderating effects

## Abstract

Food safety has received unprecedented attention since the COVID-19 outbreak. Exploring food safety regulatory mechanisms in the context of cluster public crises is critical for COVID-19 prevention and control. As a result, using data from a food safety regulation survey in the Bei-jing-Tianjin-Hebei urban cluster, this paper investigates the impact of food safety regulation on the prevention and control of COVID-19. The study found that food safety regulation and cluster public crisis prevention and control have a significant positive relationship, with the ability to integrate regulatory resources acting as a mediator between the two. Second, industry groups argue that the relationship between regulatory efficiency and regulatory resource integration should be moderated in a positive manner. Finally, industry association support positively moderates the mediating role of regulatory re-source integration capacity between food safety regulatory efficiency and cluster public crises, and there is a mediating effect of being moderated. Our findings shed light on the mechanisms underlying the roles of regulatory efficiency, resource integration capacity, and industry association support in food safety, and they serve as a useful benchmark for further improving food safety regulations during the COVID-19 outbreak.

## Introduction

The number of foodborne disease incidents and patients in China increased by 1–2 times between 2012 and 2017, raising public concern about foodborne public crises prevention and management. This concern has been heightened by the COVID-19 outbreak (1, 2). One of the most important measures in the fight against the COVID-19 outbreak is food safety regulation, which is an important component of national governance systems (3). The government has prioritized the rationalization of various food safety regulatory inputs to reduce the spread of COVID-19 in the food and retail sectors during the 2019 corona-virus disease epidemic (4, 5). Despite evolving and improving regulations related to biosecurity, hygiene standards, disease surveillance, and monitoring methods (6), poor enforcement and a lack of control over wildlife food safety contributed to the outbreak and rapid spread of the initial COVID-19 outbreak (7, 8).

Keeping a close eye on food safety was one of the most effective ways to control the COVID-19 epidemic during the epidemic of the novel coronavirus (9, 10). As a result, consumer attitudes toward food safety have shifted, and the general public is becoming more concerned about, and trusting, government food safety information (11, 12). Although COVID-19 does not appear to be a foodborne disease, it has been linked to food in some outbreaks, which may have an impact on government food control practices (13–15). The COVID-19 outbreak has in-creased public awareness of food safety, hastening the dissemination of food safety related information, and has made it easier to prevent foodborne disease outbreaks (16, 17).

COVID-19 has had a significant impact on the global economy, with the restaurant and hospitality tourism industries among the hardest hit (9, 15). A more resilient food safety regulatory system is required to help the restaurant and tourism industries recover as quickly as possible (18), as well as to respond more effectively and appropriately to large-scale incidents (3, 19). Despite the low risk of COVID-19 transmission through food, government officials do not prioritize COVID-19 tracking in the workplace (20, 21). Therefore, A well-developed food safety system is critical to responding to the epidemic challenge (22). Examining the mechanisms by which food safety regulation works to combat cluster public crises can aid in the recovery of the commercial catering and food-related industries from the severe effects of COVID-19 while also contributing to social safety and stability (23).

Based on the foregoing, this paper may contribute to existing research in the following ways: First, this paper reveals the mechanism of the role of food safety on cluster public crisis prevention and control by analyzing the relationship between the efficiency of food safety regulation and the effectiveness of cluster public crisis prevention and control, providing a strong reference for the COVID-19 epidemic prevention and control. Second, previous food safety research has primarily focused on the role of the government as an administrative body, ignoring the role of market participants such as businesses and industries. This paper introduces industry association support indicators and proposes a food safety regulation model that works in tandem with the government and the market. Finally, traditional food safety research focuses solely on the development of a preliminary food safety regulatory system or the prevention and control of post-event outcomes, with no comprehensive analysis. This paper provides theoretical guidance for the prevetion and control of the COVID-19 epidemic, as well as a comprehensive evaluation system for food safety pre-regulation and post-control.

## Literature review and hypothesis development

### Food safety regulatory efficiency and public crisis prevention effectiveness

The efficiency of food safety regulation is defined as the ratio relationship between regulatory inputs and regulatory benefits (24, 25). The most common regulatory input elements are economic input indicators, technical support, safeguard measures, and administrative regulations (26–29). Furthermore, major relevant output factors are the product quality qualification rate, food company quality and safety compliance rate, and lastly, the public food safety awareness (30–32).

Regulatory funding intensity has a positive effect on regulatory efficiency improvement in terms of economic input, more specifically, the lower the output of foodborne diseases, the more efficient food safety regulation will be (33, 34). Regarding to technology, the use of emerging technologies can assist in the assurance of food safety and quality (35, 36). The primary goal of biometric technology and its application system is to control public health risks through physiological and behavioral identification (37). Accelerating the development of biometric technology and relevant application systems, on the one hand, can facilitate multiple parties working together in order to respond to public health emergencies. On the other hand, the development of biometric technology can also improve the development of national biosafety risk assessments (38). Collaboration among governmental authorities, scientific institutions, and the general public can improve regulatory effectiveness and create a “problem-oriented” food safety regulatory system which is capable of adapting to prior risk prevention model, and is advantageous in terms of security (39, 40). Establishing and improving regulations related to food production, transportation, and operation can effectively reduce food safety violations, strengthen social management and control of food safety crimes, and lastly, reduce the possibility of public food safety violations (39). Consequently, there is a significant positive relationship between the efficiency of food safety regulation and the effectiveness of preventing and controlling mass public crises. More specifically, the higher the efficiency of food safety regulation, the better the effectiveness of preventing and controlling mass public crises will be. Based on the preceding analysis, the following hypotheses are proposed.

*Hypotheses 1: The efficiency of food safety supervision and the prevention and control of mass public crises are positively correlated*.

### The mediating role of food safety integration capacity

The abilities to identify opportunities, select partners, match resources, and manage risk are critical parts of the integration capability (41, 42). In addition, security technology is combined with current organizational integration capabilities (43). Furthermore, food safety information integration capabilities include demand identification, resource allocation, activity coordination, risk prevention, and control measure optimization (44). Therefore, food safety integration capacity includes several aspects such as regulatory requirements, resource allocation, coordination of participating actors, and optimization of risk prevention and control (45, 46). Financial assistance and incentives from the government and other foundations may inspire the interest of organizations governing food safety (41). In the meanwhile, food safety can achieve good governance only when the profits of law-abiding food producers and operators exceed the illegal income, similarly, when the illegal losses exceed the cost of following the law (47, 48).

As a result of technological advancements, the ability to integrate food safety resources has improved, lowering the risk of food safety incidents further (49, 50). However, the lack of corporate social responsibility and risk awareness, as well as backward processing technology, have made food safety supervision more difficult (51, 52). Therefore, it is critical to allow companies to participate in food safety supervision in order to make production process information more transparent, and further allowing continuous product quality improvement (53–55). Finally, all four dimensions of regulatory efficiency have a positive impact on food safety integration capacity. More specifically, increasing food safety integration capacity aids in the prevention of cluster public crises. Consequently, the following hypotheses are constructed.

*Hypotheses 2: The ability to integrate food safety regulatory resources mediates the relationship between regulatory efficiency and cluster public crisis prevention and control*.

### The regulating role of industry association support

Since food safety is a highly complex issue involving multiple sectors, policies, as well as being clearly cross-border in its nature (56), industry associations should participate and support food safety activities (57). Food safety can only be governed effectively by integrating relevant government authorities, market, social forces, as well as communication mechanisms in order to form common goals, norms, action plans, and by recognizing and complying multiple parties (57–59). Hence, the active participation and promotion of industry associations play a critical role in improving the efficiency of food safety supervision (31, 60). Despite the fact that rural China has developed a diverse model of food security governance, this model is not yet well defined (61). If the government takes the lead without participation and promotion of industry associations, it is difficult for a pluralistic governance model to be established (62).

As regulatory efficiency improves, it becomes more important to regulate and improve food company integration (63). Consequently, the establishment of a dynamic food safety regulatory system will assist the government in tracking and assessing the self-regulatory behavior of food safety companies, as well as in timely adjusting regulatory strategies, improving regulatory efficiency (64). In addition to being driven by the government, these regulations necessitate the active participation and support of industry associations (65). External pressures on food companies to meet their food safety obligations can effectively reduce the food industry's regulatory burden (66, 67). Simultaneously, in some cases, reputational mechanisms, in addition to external pressures, can provide incentives for companies to provide safe and high-quality food (68). Building and maintaining a regional food reputation necessitates the ability to bring various producers' interests together (69). Therefore, increasing trade association organization is critical for improving regulatory effectiveness by integrating government resources and developing a flexible regulatory system (70). In other words, increasing the effectiveness of cluster public crisis prevention and control by leveraging the role of market players to improve food safety integration with the help of industry associations becomes more important. As a result of this, the following hypothesis is proposed.

*Hypothesis 3: The support of Industry association moderates the relationship between regulatory efficiency and the ability to integrate regulatory resources*.

*Hypothesis 4: The support of Industry association positively moderates the ability to integrate regulatory resources and the prevention of public crises*.

Industry associations' support provides the necessary assurances and creates a favorable regulatory environment (71, 72). Various regulatory efficiency mechanisms and specific initiatives increase the capacity for food safety regulatory integration, lowering the likelihood of cluster public crises (73, 74). The above-mentioned mediating role of food safety integration capacity, as well as the two-stage moderating role of industry association support, show that regulatory efficiency influences the effectiveness of cluster public crisis prevention and control *via* the mediating role of food safety integration capacity (75), an effect that is aided by industry association support (76). Therefore, in the pathway “regulatory efficiency—food safety integration capacity—cluster public crisis prevention,” the mediating effect of industry association support on food safety integration capacity is moderated (77, 78). As a result, the following hypotheses are proposed.

*Hypothesis 5: Industry associations, through their ability to integrate regulatory resources, play a mediating role in positively regulating the impact of regulatory efficiency on cluster public crises*.

**Figure 1:**
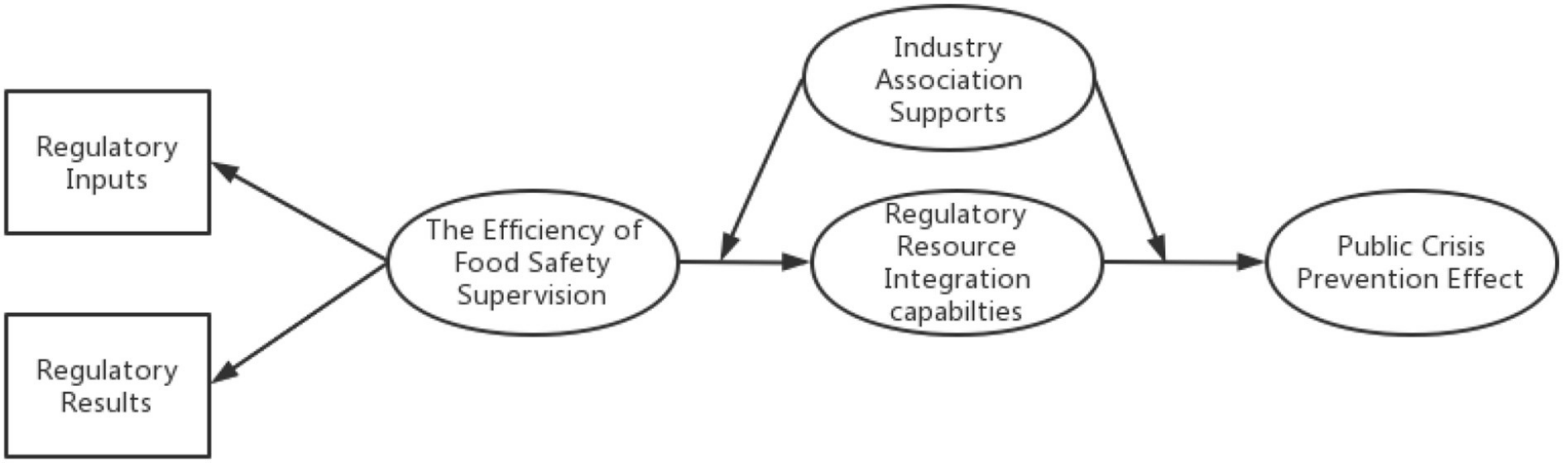
Path diagram of the empirical study.

We propose the following empirical research path model diagram (Figure 1).

## Research design

### Study sample and data sources

This paper examines the impact of food safety supervision efficiency on the effectiveness of cluster public crisis prevention and control using 7-year input-output panel data from 2015 to 2021 across 32 districts in Beijing and Tianjin, as well as 11 prefecture-level cities in Hebei Province. Relevant data was collected and complied from the Food Safety Partnership, the Food and Drug Supervision Statistics Annual Report, the Health Commission of Hebei Provincial, the Health Commission of Beijing, and the Health Commission of Tianjin. Questionnaires were used to collect information on the variables of food safety integration, industry association support, and cluster public crisis prevention effectiveness.

The survey sample size was determined by random sampling and the process of its determination was as follows:


(1) $$\begin{array}{l} n=Z^{2}\times \sigma^{2}/d^{2} \end{array}$$

*n* represents the required sample size and *Z* represents the *z*-statistic at 90% confidence level (*Z* = 1.64). σ represents the standard deviation of the overall population and takes the value of 0.5. *d* is 1/2 of the confidence interval, which in practical terms is the tolerance error, or survey error. The final number of questionnaires placed was determined to be 269.

The survey results were 35 for Tianjin citizens, 56 for Beijing citizens and 49 for Hebei citizens. There were 22 staff members of the Tianjin Municipal Administration of Market Supervision, 37 staff members of the Hebei Provincial Administration of Market Supervision and 34 staff members of the Beijing Municipal Administration of Market Supervision. Twenty two postgraduate students in food science and safety engineering (11 for Ph.D. and 11 for M.Sc.). A total of 255 valid questionnaires were collected and 14 invalid questionnaires were returned, accounting for 5% of the invalid questionnaires and meeting the 90% confidence interval required for the survey. The main reason for the invalid questionnaires was that the survey population did not live in the Beijing-Tianjin-Hebei region for more than 30 days.

### Relevant variable indicators and measurements

Independent variable: food safety regulatory efficiency. By analyzing the inputs and outputs of food safety regulation, the DEA model was used to analyse food safety efficiency, and the results of the analysis of food safety regulation efficiency were used as the independent variable to participate in the analysis model. The evaluation indicators of food safety regulatory efficiency were based on the evaluation indicators of Kokkinakis et al. (79) and Varzakas et al. (80), which were modified to take into account the availability of data. Considering that there does not exist a fixed function model between the input and output indicators of food safety supervision efficiency, and the DEA model effectively circumvents this problem by optimizing the process to determine the weights of input and output variables.

The evaluation indicators related to the efficiency of food safety supervision (as shown in Table 1) include two primary indicators of input and output. The input indicators include four secondary indicators: economic input, human input, safeguard measures and administrative input. Economic input directly reflects the importance attached to food safety regulation, which is reflected through financial investment and investment in technological research and development. Human input reflects the level of investment in food safety supervision, as expressed by the number of supervisors and R&D staff. Safeguarding measures reflect the investment in safeguarding food safety regulation, as measured by the number of honest food enterprises and the number of industry associations. Administrative inputs show the most direct government input into food safety, measured by the intensity of food sampling and food safety regulations. The output variable contains three aspects: food safety, public reaction and business operations. Food safety output is measured by the reporting of major food safety incidents and the passing rate of product sampling and inspection. Public response is measured by the number of foodborne illnesses. Business operation is expressed through the QS compliance rate of food enterprises.

**Table 1:** Food safety supervision efficiency evaluation index system.

**First indicators**	**Secondary indicators**	**Third indicators**	**Unit**
Input indicators	Economic inputs	Investment in regulatory funding	Million
		Investment in R&D of regulatory technology	Million
	Human input	Number of supervisory personnel	Person
		Regulatory technology research and development personnel	Person
	Safeguard measures	The number of honest food enterprise	Number
		Number of industry associations	Number
	Administrative input	Food sampling and inspection efforts	Times
		Food safety administrative regulations	Items
Output indicators	Food Safety	Number of reported major food safety accidents	Piece
		Product sampling and inspection pass rate	%
	Public response	Number of food-borne diseases	Person
	Enterprise operation	QS compliance rate of food enterprises	%

Charnes et al. (81) enriched the concept of multiple inputs and outputs proposed by Farrell (82) into the CCR model with constant returns to scale and the BCC model with variable returns to scale. For the study sample it is assumed that there are N decision making units (DUM), each of which has Z input variables *X*_*i*_ = (*X*_*i*1_, *X*_*i*2_, …, *X*_*im*_), where *iε*[1, *Z*]. There are also W output variables *Y*_*j*_ = (*Y*_*j*1_, *Y*_*j*2_, …, *Y*_*jm*_), where *jε*[1, *W*]. Since the underlying assumption of the C2R model is that the payoff to scale is constant, and in practice various factors can affect the efficiency of food safety regulation, the BCC model with variable payoff to scale, obtained by supplementing the CCR model with non-Archimedean infinitesimals, is chosen (81, 83).


(2) $$\left\{ \begin{array}{c} min[\alpha -\upsilon ({\sum^{\text{​}}}_{i=1}^{Z}S^{-}+{\sum^{\text{​}}}_{j=1}^{W}S^{+})]\\ {\sum^{\text{​}}}_{p=1}^{n}\lambda_{p}\;x_{ip}+S_{i}^{-}=\alpha x_{if},\;i\in [1,Z]\\ \begin{array}{c} s.t.{\sum^{\text{​}}}_{p=1}^{n}\lambda_{p}\;y_{jp}-S_{j}^{+}=y_{jf},j\in [1,W]\\ {\sum^{\text{​}}}_{p=1}^{n}\lambda_{p}=1,p\in [1,n]\\ \;\lambda_{p},\alpha ,S_{i}^{-},S_{j}^{+}\geq 0,p\in [1,n] \end{array} \end{array}\right.$$

where *x*_*if*_, *y*_*if*_ denotes the *i*-th input and *j*-th output of the *f*-th decision unit (DUM_*f*_), respectively. Similarly, *x*_*ip*_, *y*_*ip*_ denotes the *i*-th input and *j*-th output of the *p*-th decision unit (DUM_*p*_), $S_{j}^{+}$ is the residual variable, $S_{j}^{-}$ is the slack variable, λ_*p*_ is the weight, υ is the non-Archimedean infinitesimal, and α is the pure technical efficiency. When *TE* = *1, that is, PTE* = *1, SE* = *1*, the integrated technical efficiency (*TE*) in *BCC* is divided into pure technical efficiency (*PTE*) and scale technical efficiency (*SE*), *TE* = *PTE* × *SE*, and the decision unit (*DUM*) is effective. Because the *DEA* model for input-output indicators requires positive values, and economic input indicators are frequently large, the data are dimensionless to normalize them to the interval [0.1, 1], and equation (2) is used for data processing.


(3) $$\begin{array}{l} {\text{y}}_{\text{ij}}=\text{0}.\text{1}+\frac{x_{ij}-m_{ij}}{M_{ij}-m_{ij}}\times 0.9 \end{array}$$

Among them, *m*_*ij*_ = min*x*_*ij*_, *M*_*ij*_ = max*x*_*ij*_, *y*∈[0.1, 1], *i*∈(1, *n*).

The capacity for food safety integration is the mediating variable. The Vanpoucke et al. (84) scale was modified to assess food safety integration capacity. Seven distinct questions assess the government's ability to identify people's food safety needs, rationally allocate resources, coordinate the participation of various actors (for example, companies, schools, and individuals), and prevent and control foodborne illness.

The moderating variable—industry association support. The industry-supported measure is based on Nakku et al.'s (85) revised scale and consists of five questions designed to assess industry associations' awareness and involvement in food safety management (85).

Dependent variable—Effectiveness of cluster public crisis prevention and control. Christensen et al.'s (86) revised scale, which consists of seven questions designed to assess the effectiveness of the government's overall food safety prevention and control, is used to assess the effectiveness of cluster public crisis prevention and control (86).

Control variables-enterprise size and level of industry development. The scale and level of enterprise development are frequently linked to the integrity of food safety and the food safety measures implemented. Food safety may be valued differently by different types of businesses. Existing research indicates that the scale of enterprise development is positively correlated with the level of food safety management, implying that the larger the scale of enterprise, the greater the emphasis on food safety will be. The information was gathered from three regional statistics bureaus and included the number of food enterprises as well as the total value of the food industry in the Beijing-Tianjin-Hebei region. The level of development of an industry is frequently linked to the level of economic development due to its uniqueness, whereas the food industry is frequently linked to people's consumption levels.

## Empirical results

### Beijing, Tianjin and Hebei food safety supervision efficiency

The DEAP 2.1 software was used to measure the efficiency of food safety supervision in 43 urban areas in Beijing, Tianjin and Hebei from 2015 to 2019. CRS, VRS and SE denote comprehensive technical efficiency, pure technical efficiency and scale technical efficiency, respectively, and the ratio of CRS to VRS is SE.

As shown in Table 2, the combined technical efficiency CRS is the production efficiency of certain input factors, and is an evaluation of the combined ability of various aspects such as the ability to allocate resources and the efficiency of resource use (87). The average value of the combined technical efficiency for the seven years was 0.916, with only five regions reaching a combined technical efficiency of 1, i.e., effective regulation. Pure technical efficiency VRS is the efficiency brought about by the level of institutions and management, which is the productive efficiency due to factors such as management and technology. The mean value of pure technical efficiency for the 7 years is 0.926, which is relatively high, with only 7 regions reaching 1 in pure technical efficiency, accounting for 21.2% of the sample capacity. As pure technical efficiency largely reflects the level of regional management, regions whose development is already more mature and those in the rapid growth stage have stronger regulatory management capabilities. Examples include Beijing's Dongcheng and Xicheng districts as well as Haidian District; Tianjin's Heping and Nankai districts; and Shijiazhuang and Xiongan New Area in Hebei Province. Meanwhile, the scale efficiency SE reflects the influence of regional scale factors on regulatory efficiency; the mean value of the scale effect is 0.926 over the seven-year period, which is relatively high. In particular, Haidian District in Beijing, Heping District and Nankai District in Tianjin, and Shijiazhuang City in Hebei Province. In addition, Cangzhou City has the lowest overall technical efficiency and pure technical efficiency; meanwhile, Chengde City and Hengshui City have lower overall technical efficiency, pure technical efficiency and scale efficiency. This indicates that these three regions have improved their resource investment, systems and management capacity in food safety supervision, and the scale of regional development needs to be further improved.

**Table 2:** Efficiency of food safety supervision in cities and districts in Beijing-Tianjin-Hebei.

**City, district**	**CRS**	**VRS**	**SE**	**City, district**	**CRS**	**VRS**	**SE**
Dongcheng	0.994	1	0.994	Dongli	0.888	0.863	1.030
Xicheng	0.948	1	0.948	Xiqing	0.972	0.934	1.041
Chaoyang	1	0.992	1.008	Jinan	0.917	0.941	0.974
Fengtai	0.945	0.992	0.953	Beichen	0.913	0.965	0.946
Shijingshan	0.977	0.916	1.067	Wuqing	0.821	0.823	0.998
Haidian	1	1	1	Baodi	0.976	0.922	1.059
Mentougou	0.918	0.886	1.036	Binhai	0.956	0.943	1.014
Fangshan	0.872	0.844	1.033	Ninghe	0.888	0.876	1.014
Tongzhou	0.894	0.924	0.968	Jinghai	0.985	0.963	1.023
Shunyi	0.87	0.885	0.983	Jizhou	0.967	0.943	1.025
Changping	0.911	0.844	1.079	Shijiazhuang	1	1	1
Daxing	0.905	0.914	0.990	Tangshan	0.955	0.964	0.991
Huairou	0.953	0.951	1.002	Qinhuangdao	0.965	0.976	0.989
Pinggu	0.908	0.841	1.080	Handan	0.884	0.842	1.050
Miyun	0.862	0.943	0.91	Baoding	0.891	0.911	0.978
Yanqing	0.946	0.922	1.03	Zhangjiakou	0.876	0.909	0.964
Heping	1	1	1	Chengde	0.706	0.876	0.806
Hexi	0.969	0.997	0.972	Cangzhou	0.668	0.687	0.972
Hedong	0.966	0.934	1.034	Langfang	0.823	0.913	0.901
Nankai	1	1	1	Hengshui	0.679	0.814	0.834
Hebei	0.939	0.997	0.942	Xiong'an	0.946	1	0.946
Hongqiao	0.921	0.966	0.953	Mean	0.916	0.926	0.989

### Common method deviation analysis

In the process of empirical research based on questionnaires, questionnaire results frequently lead to common method bias due to a variety of factors such as subject source, questionnaire question characteristics, questionnaire content bias, and measurement environment bias. As a result, common method bias was examined for 255 questionnaires in this paper. In order to address how these biases impact research results effectively, this paper chose the potential error variable control method and constructed a factorial model to analyze common method bias as a variable of the model. In order to fit the model properly, this paper uses AMOS 24.0 software for common method bias analysis, and it is also necessary to set a qualification for the common method bias latent variable and set the variance of the common method bias latent variable to 1. The results are shown in Table 3. Root mean square of the residuals (RMR), which is the sum of the squares of the sample variances and covariances minus the corresponding estimated variances and covariances, and then the square root of the mean. RMR should be <0.08, the smaller the RMR, the better the fit. Root Mean Square Error of Approximation (RMSEA), which should be <0.06, the smaller the better. The Goodness of Fit Index (GFI), which ranges between 0 and 1, but can theoretically produce negative numbers that are not meaningful. As a rule, to accept the model, the GFI should be =/>0.90. The simple goodness of fit index, PGFI, is the simple ratio (PRATIO, the ratio of the degrees of freedom of the independent model to the degrees of freedom of the internal model) multiplied by the GFI. The PGFI should be =/>0.90, with the closer to 1 the better. Parsimonious goodness of fit index (PNFI), equal to PRATIO multiplied by NFI. the PNFI should be =/>0.90, the closer to 1 the better. Normative fit index (NFI), varies between 0 and 1, 1 = perfect fit. An NFI of <0.90 indicates that the model needs to be reset. Closer to 1 is better. Comparative fit index (CFI) with values between 0 and 1. When CFI close to 1 indicates a very good fit and a value above 0.90 indicates an acceptable model, the closer to 1 the better.

**Table 3:** Analysis of common method deviations.

	***x*** **^2^**	**RMR**	**RMSEA**	**GFI**	**PGFI**	**NFI**	**NNFI**	**CFI**
Model without common method deviation	1.339	0.073	0.07	0.903	0.913	0.958	0.934	0.976
Model with common method deviation	1.529	0.079	0.079	0.907	0.915	0.961	0.923	0.958

After adding the common method bias latent variable, the fit index of the model increased by 0.19 and the RMSEA index increased by 0.009, but CFI and NNFI decreased by 0.018 and 0.011, respectively, indicating that the fit index of the model did not improve and proving that there was no significant common method bias.

### Reliability and validity tests

The reliability and validity of the questionnaire were evaluated using indicators of combined validity and factor loadings, while the structural validity was evaluated using validation factor analysis. Relevant results are shown in Table 4. The test of discriminant validity was evaluated using the square root of the latent variable AVE indicator and the correlation value between the latent variables.

**Table 4:** Reliability and validity tests of the questionnaire measurement scales.

**Variables**	**Measurement issues**	**Factor loading**	**AVE**	**CR**	**Cronbach's α**
Food safety regulatory efficiency	Food safety funding investment can improve regulatory efficiency	0.939	0.769	0.881	0.893
	Food safety regulatory technology research and development investment can improve the efficiency of regulatory washing	0.776			
	The more supervisory personnel the higher the efficiency of food safety supervision	0.869			
	The more R&D personnel of regulatory technology the higher the efficiency of food safety regulation	0.723			
	The more the number of honest food enterprises, the higher the efficiency of food safety regulation	0.669			
	The more the number of industry associations the higher the efficiency of food safety regulation	0.730			
	The greater the intensity of food sampling and inspection the higher the efficiency of food safety supervision	0.988			
	The more administrative food safety regulations, the more efficient food safety regulation	0.910			
Ability to integrate regulatory resources	The government is able to identify the food safety needs of the people	0.885	0.723	0.894	0.937
	The government is able to allocate food safety resources appropriately	0.763			
	The government is able to promote good cooperation between the actors in the market (e.g., enterprises, universities, NGOs) and to improve the level of cooperation and synergy between them	0.896			
	Governments can coordinate the food safety activities of different enterprises	0.747			
	Governments educate about food safety risks and preventive measures	0.913			
	Government is able to share information and knowledge about food safety	0.956			
	Government is able to implement safety rating programs for food businesses	0.823			
Industry association support	Industry associations are interested in advancing food safety related issues	0.813	0.593	0.811	0.767
	Specific actions of industry associations convey the importance of food safety	0.688			
	Industry associations support the implementation of food safety management systems	0.864			
	Access to food safety information published by industry associations	0.634			
	Industry associations share the same goals as citizens regarding food safety	0.573			
	Services that industry associations can provide regarding food safety	0.493			
The effect of preventing mass public crises	Government's ability to manage public crises, quality of governance, efficiency and management has improved	0.913	0.719	0.800	0.919
	Timeliness of government public crisis services has improved	0.921			
	The regional government has a clear advantage in the management of public crises	0.864			
	The government's ability to manage public crises and the measures taken to manage them match the expectations of the public	0.893			
	The effectiveness of public crisis prevention and control has increased citizens' confidence in the government's crisis prevention and control	0.883			
	The establishment of a public crisis prevention and control system has improved the public crisis prevention and control system	0.863			

Table 4 shows that the combined reliability of food safety regulatory efficiency, regulatory resource integration ability, industry association support, and public crisis prevention and control effect is 0.881, 0.894, 0.811, and 0.8, which are all >0.7. The AVE values for latent variables are 0.769, 0.723, 0.593, and 0.719, all of which are >0.5. Cronbach α's coefficients for the four latent variables were all >0.7: 0.893, 0.937, 0.767, and 0.919. In the meantime, the square root of the AVE of the four latent variable points in Table 5 was greater than their correlation coefficients with one another, indicating that the measurement scale was reliable and valid.

**Table 5:** Mean, variance and square root of AVE for latent variables.

	**1**	**2**	**3**	**4**
Regulatory efficiency	0.769			
Integrated ability	0.686	0.723		
Industry association support	0.631	0.667	0.593	
Control effect	0.598	0.638	0.569	0.719

### Hypothesis testing

Table 6 displays descriptive statistics, including means, standard deviations, and correlation coefficients, for the four variables of food safety regulatory efficiency, regulatory resource integration capacity, industry association support, and public crisis prevention and control effectiveness.

**Table 6:** Means, standard deviations and correlation coefficients.

	**Average value**	**SD**	**1**	**2**	**3**	**4**
Regulatory efficiency	0.881	0.596	—			
Integrated ability	0.836	0.667	0.659^***^	—		
Industry association support	0.769	0.589	0.268^**^	0.501^*^	—	
Control effect	0.913	0.684	0.681^***^	0.607^**^	0.612^***^	—

*, **, *** are significant at 0.1, 0.05, and 0.01 respectively.

Between food safety regulatory efficiency and food safety resource integration capacity, a significant positive correlation (*R* = 0.659, *P* < 0.01) was discovered. A significant positive correlation (*R* = 0.607, *P* < 0.05) was discovered between regulatory resource integration capacity and public crisis prevention and control effect. f Food safety regulatory efficiency had a significant positive correlation with public crisis prevention and control effect (*R* = 0.681, *P* < 0.01), as well as a positive correlation with industry association support (*R* = 0.268, *P* < 0.05). The positive correlation between food safety supervision efficiency and industry association support (*R* = 0.268, *P* < 0.05) shows that industry associations play a minor role in food safety supervision in the Beijing-Tianjin-Hebei region, and that the government continues to dominate the system. There is a positive correlation between supervisory resource integration ability and industry association support (*R* = 0.501, *P* < 0.1); there is a positive correlation between industry support and public crisis prevention and control effect (*R* = 0.612, *P* < 0.01). Table 7 displays the results of stepwise regression hypothesis testing performed using SPSS24.0 software to validate the research hypotheses.

**Table 7:** Stepwise regression results.

	**Dependent variable**			**Dependent variable**			
	**Integrated ability**			**Control effect**			
	**Model 1**	**Model 2**	**Model 3**	**Mode 4**	**Model 5**	**Model 6**	**Model 7**
**Control variables**							
Industry development level	0.138^*^	0.103	0.097	0.146^*^	0.117^**^	0.107^*^	0.089^***^
Enterprise size	0.201^**^	0.161^*^	0.147	0.186^**^	0.126^*^	0.155^**^	0.143^*^
**Independent variables**							
Regulatory efficiency		0.327^*^	0.229^**^		0.461^**^	0.403^*^	
**Intermediary variables**							
Integrated ability						0.371^**^	0.336^***^
**Moderating Variables**							
Industry association support			0.137^**^				0.173^**^
**Interaction term**							
Regulatory efficiency × industry association support		0.115^*^				
Integrated ability × industry association support			0.068
*r* ^2^	0.053	0.122	0.216	0.098	0.336	0.267	0.376
Δ *r*^2^	—	0.103	0.135	—	0.138	0.147	0.109

*, **, *** are significant at 0.1, 0.05, and 0.01 respectively.

To test the relationship between food safety regulatory efficiency and the effectiveness of cluster public crisis prevention and control, a regression was conducted with the effectiveness of public crisis prevention and control as the dependent variable and the industry development level and enterprise size as the independent variables. Model 4 displays the outcomes. Meanwhile, model 5 adds the independent variable of food safety regulation efficiency based on model 4, and the regression results show that food safety regulation efficiency has a significant positive effect on public crisis prevention and control (α = 0.461, *P* < 0.05). Therefore, the hypothesis 1 was proved correct.

The dependent variable-public crisis prevention and the independent variable-food safety supervision efficiency are used to test the mediating effect of regulatory resource integration ability. The results are shown in Model 5, with a coefficient of food safety supervision efficiency of 0.461 (*P* < 0.05). Secondly, regulatory integration ability is the dependent variable, while the independent variables are industry development level, enterprise size, and food safety supervision efficiency. Models 1 and 2 both display the results. Model 1 results show that, with coefficients of 0.138, *P* < 0.1 and 0.201, *P* < 0.05, respectively, industry development level and enterprise size can effectively explain Model 1. Simultaneously, the efficiency of food safety supervision has a significant positive impact on the ability to integrate supervision resources (α = 0.371, *P* < 0.05). Finally, the dependent variable is the effect of public crisis prevention, while the independent variables are the efficiency of food safety supervision and the ability of supervision resource integration. Model 6 displays the outcomes. Food safety supervision efficiency has a significant positive effect on public crisis prevention, with a coefficient of 0.403, which is significant at the level of 0.1. The ability to integrate regulatory resources has a positive impact on the public crisis prevention effect (α = 0.371, *P* < 0.05). When the results of Model 5 and Model 6 are compared, the significance level of food safety supervision efficiency decreases, as does the coefficient value (0.461 > 0.403). As a result, hypothesis 2 is supported: the impact of food safety supervision efficiency on the prevention and control effect of public crises is mediated in part by the ability of supervision resources to integrate.

Model 3 shows the regression of regulatory resource integration ability as the dependent variable and the independent variables of industry development level, enterprise size, food safety regulatory efficiency, industry association support, and the interaction term of food safety regulatory efficiency and industry association support, as well as the interaction term of food safety regulatory efficiency and industry association support, to test the moderating effect of industry association support. Model 3 displays the outcomes. There is an interaction term between food safety regulatory efficiency and industry association support. Model 3 demonstrates that the interaction term of food safety regulatory efficiency and industry association support has a significant positive effect on the ability to integrate regulatory resources (α = 0.115, *P* < 0.1), indicating that the positive moderating effect of industry association support on food safety regulatory efficiency and the ability to integrate regulatory resources is significant and hypothesis 3 is supported. The dependent variable was the public crisis prevention and control effect, with the independent variables being the industry development level, enterprise scale, regulatory resource integration capability, industry association support, regulatory resource integration capability, and industry association support interaction terms. Model 7 displays the results of the regression after it has been run. The interaction term supported by regulatory resource integration capabilities and industry associations has no effect on the public crisis prevention effect (α = 0.068, *P* > 0.1). The fourth hypothesis has yet to be proven. The objective facts are also consistent. In China, the government currently controls the majority of food supervision. In the integration of supervision resources, the government is the primary body. At the same time, relevant food industry associations in my country are still in their infancy, with little impact on the integration of supervision resources. We examine the mediation effects supported by various levels of industry associations using the process plug-in in SPSS24.0 to test hypothesis 5. The results of verifying the mediation variable's effect size and significance level are shown in Table 8.

**Table 8:** Mediation effects at different levels of industry association support.

**Adjustment variables**	**Conditional**	**0.95 confidence interval**	
	**Indirect effect**		
		**Upper limit**	**Lower limit**
Low industry association support (mean — standard deviation)	0.077	0.109	0
High industry association support (mean + standard deviation)	0.143^***^	0.221	0.091
High-low difference	0.052^**^	0.078	0.006

*,

**, *** are significant at 0.1, 0.05, and 0.01 respectively.

The results show that food safety supervision efficiency has a significant positive impact on the prevention and control effect of public crises *via* supervision resource integration ability (α = 0.143, *P* < 0.01), with a confidence interval of 0.95 [0.091, 0.221] under high levels of industry association support (α = 0.143, *P* < 0.01). When industry association support is low, the impact of food safety supervision efficiency on the prevention and control effect of public crises through the ability of supervision resource integration is not significant (α = 0.077, *P* > 0.1), and the confidence interval of the indirect effect is 0.95 [0, 0.109]. Simultaneously, with a 0.95 confidence interval of [0.006, 0.078], the difference in indirect effects supported by industry associations at various levels reached significance (α = 0.052, *P* < 0.05). As a result, strong industry support will moderate the ability of regulatory resources to integrate, resulting in a moderated mediation effect. Hypothesis 5 has been verified.

### Robustness tests

This paper examines 255 questionnaires and food safety supervision input and output data from the Beijing, Tianjin, and Hebei regions separately to test the robustness of the research model, with the results shown in Table 9. In comparison to Table 6, the model in the significance level of the verification of the results for the change, only in the specific value of the change size. As a result, both the research sample and the model in this paper are extremely robust.

**Table 9:** Robustness test results.

	**Dependent variable**			**Dependent variable**			
	**Integrated ability**			**Control effect**			
	**Model 1**	**Model 2**	**Model 3**	**Mode 4**	**Model 5**	**Model 6**	**Model 7**
**Control variables**							
Industry development level	0.116^*^	0.097	0.063	0.109^*^	0.109^**^	0.094^*^	0.077^***^
Enterprise size	0.153^**^	0.481^*^	0.116	0.145^**^	0.104^*^	0.108^**^	0.120^*^
**Independent variables**							
Regulatory efficiency		0.219^*^	0.157^**^		0.398^**^	0.363^*^	
**Intermediary variables**							
Integrated ability						0.307^**^	0.291^***^
**Moderating Variables**							
Industry association support			0.101^**^				0.122^**^
**Interaction term**							
Regulatory efficiency × industry association support		0.095^*^				
Integrated ability × industry association support		0.043
*r* ^2^	0.059	0.134	0.199	0.102	0.341	0.249	0.358
Δ *r*^2^	—	0.099	0.118	—	0.117	0.169	0.132

*, **, *** are significant at 0.1, 0.05, and 0.01 respectively.

## Discussion

### Theoretical contribution

The audience will gain a better understanding of the effectiveness of food safety regulations and the prevention of large-scale public safety crises as a result of the findings. The improvement of administrative legislation and enforcement, as well as the related policy effects, are intuitively reflected in food safety regulation (4, 8, 20, 28, 47). As a result, previous research has concentrated on evaluating food safety regulation policies, administrative legislation, administrative enforcement, and law enforcement. While there has been less research on specific factors such as the social benefits of food safety regulations, infrastructure, technological progress, factor accumulation, institutional environment, talent attractiveness, economic scale, and regional heterogeneity, there has been some (10, 54, 68, 74, 78). Existing research primarily concerned with the social benefits of cities that have well-developed food safety supervision, with few studies focus on how to establish a food safety supervision system suitable for national development which can alleviate existing constraints and improve the ability to prevent mass public crises. In order to be consistent with this new research direction, our research broaden the scope of the discussion to include the development history of the food safety regulatory system (3, 11, 26, 67, 74). This study also combines the efficiency of food safety supervision in the Beijing-Tianjin-Hebei region with academic research on mass public safety crises. The DEA model is used to measure food safety supervision efficiency and test the effect of food safety supervision efficiency on the prevention and control of mass public crises empirically based on the calculation results.

Secondly, our findings emphasize the importance of promoting food safety oversight. According to previous research on the construction of a food safety supervision system, the impact of food safety supervision on mass public safety crises is reflected in the synergy and trade-off relationship between different entities to encourage the development of food safety supervision (28, 49, 59). In this article, we concentrate on various elements that affect food safety supervision since they are critical for maximizing the food safety supervision system's effectiveness in preventing mass public crises. Our research highlights the significance of regulatory efficiency, industry support, and the ability to integrate regulatory resources, and then deftly uncovers their mechanism for mass public crises, and lastly, promote mass public crisis governance through these mechanisms. This study explains the key aspects of the efficiency of food safety supervision and provides an effective reference for responding to the COVID-19 epidemic.

### Practical contribution

Regulatory efficiency enhancement and improvement are carried out in a differentiated manner in response to regional differences in food safety regulatory efficiency. The three regions can increase resources invested in food safety supervision, improve regional management capacity, and expand the scale of regional development to compensate for deficiencies in comprehensive technical efficiency, pure technical efficiency, and scale efficiency in Chengde City, Cangzhou City, and Hengshui City. We can increase investment in food safety supervision resources, such as human, material, and financial resources, to address deficiencies in comprehensive technical efficiency in Beijing's Huairou District, Tianjin's Wuqing District, Handan City, Zhangjiakou City, and Langfang City in Hebei Province.

Enhance your ability to integrate regulatory resources for food safety. In terms of resource integration capacity, regulatory authorities should be kept up to date on new food safety regulations on a regular basis. Rational allocation of regulatory resources, thereby optimizing risk prevention and control measures for food safety. Furthermore, to raise awareness of food safety prevention and control, as well as to coordinate food production enterprises' safety activities.

Increase the importance of industry associations as a source of assistance. On the one hand, industry associations should align their goals with those of the general public in order to align common food safety objectives; on the other hand, they should broaden the scope and influence of their food safety services in order to let the general public enjoy the results of their efforts. Industry associations, in the meanwhile, should pay more attention to food safety and participate more proactively in food safety activities, as their participation can help with food safety regulation and cluster crisis prevention, more specifically, they can help with the rational planning and design of regulatory systems and processes, provide a good environment for food safety management, and contribute to the regulation of the entire regulatory system. Integration capabilities are extremely useful.

### Limitation and future research

Some of the limitations of this paper also provide new directions for future research to be carried out. On the one hand, in the support of industry food safety regulatory resources and trade associations, a questionnaire survey was used. However, due to the limitations of the survey sample, the assessment of the results of this item may be somewhat inaccurate in relation to reality. Future research will need further evidence from a quantitative perspective using statistical methods. On the other hand, in terms of the efficiency of food safety regulation and the prevention of cluster public crises, our study uses statistics for the Beijing-Tianjin-Hebei region for the period 2015–2021. However, the Beijing-Tianjin-Hebei region, being the most developed region in China, is at a high level of economic development and technology in China, while the more backward regions are not covered in our sample.

## Conclusion

Based on the actual situation of food safety regulatory inputs and outputs in the Beijing-Tianjin-Hebei region, this paper explores the impact of food safety regulatory efficiency on the prevention and control of mass public crises, using the ability to integrate regulatory resources as a mediating variable and the support of industry associations as a moderating variable, and draws the following conclusions.

The efficiency of food safety regulation has a significant positive impact on the effectiveness of cluster public crisis prevention and control, with the ability to integrate regulatory resources playing a mediating role in the process. The power from the government and all sectors of society will promote the development of food safety regulation, creating good basic conditions for the prevention and control of major public crises. The rational allocation and coordination of regulatory resources is important for the prevention and control of major public crises, and can effectively improve the effectiveness of the prevention and control of public crises.

Industry associations support a positive relationship between the ability to integrate regulatory resources and the effectiveness of preventing and controlling mass public crises. In the process of food safety regulation, the stronger the support of industry associations, the more significant the contribution of regulatory resource integration to the prevention and control of mass public crises. The degree of attention and participation of food industry associations in food safety regulation can create a favorable environment and conditions for the prevention and control of mass public crises. The support of trade associations can help to improve the level of matching of regulatory resources with regulatory capacity, thus significantly improving the level of prevention and control of major public crises. The higher the level of support from industry associations, the stronger the mediating effect of the ability to integrate regulatory resources.

## Data availability statement

The original contributions presented in the study are included in the article/supplementary material, further inquiries can be directed to the corresponding author/s.

## Author contributions

JD: conceptualization, methodology, software, and writing—original draft preparation. PQ: validation, formal analysis, investigation, resources, and supervision. JW: data curation, writing—review and editing, supervision, funding acquisition, and visualization. HH: revising, data collection, data analysis, proofread, and investigation. All authors have read and agreed to the published version of the manuscript.

## Funding

The Ministry of Education's Humanities and Social Sciences Research Youth Fund Project Government Organization Reorganization and Enterprise Product Safety Information Disclosure-Quasi-Natural Experiment Research Based on the Newly Established Municipal Supervision Bureau (20YJC630143), National Natural Science Foundation of China Youth Project Stable Employment in the United States, Cross-border Migration of Science and Technology Talents and Innovation of Chinese Enterprises (72102229), and Hubei Province Soft Science Surface Project Research on the Technology Breakthrough of Hubei's Infrared Imaging Industry under the Crisis of Epidemic Core Shortage (2022EDA058).

## Conflict of interest

The authors declare that the research was conducted in the absence of any commercial or financial relationships that could be construed as a potential conflict of interest.

## Publisher's note

All claims expressed in this article are solely those of the authors and do not necessarily represent those of their affiliated organizations, or those of the publisher, the editors and the reviewers. Any product that may be evaluated in this article, or claim that may be made by its manufacturer, is not guaranteed or endorsed by the publisher.

## References

[1] Pal I Ghosh T Ghosh C. Institutional framework and administrative systems for effective disaster risk governance – Perspectives of 2013 Cyclone Phailin in India. Int J Disaster Risk Reduct. (2017) 21:350–9. 10.1016/j.ijdrr.2017.01.002

[2] Park J Chung E. Learning from past pandemic governance: early response and public-private partnerships in testing of COVID-19 in South Korea. World Dev. (2021) 137:105198. 10.1016/j.worlddev.2020.105198 32982017 PMC7500944

[3] Djekic I Nikolić A Uzunović M Marijke A Liu A Han J. Covid-19 pandemic effects on food safety - Multi-country survey study. Food Control. (2021) 122:107800. 10.1016/j.foodcont.2020.107800 33281304 PMC7707641

[4] Chowdhury T Nandi S. Chapter 11 - Food safety, hygiene, and awareness during combating of COVID-19. In:Hadi Dehghani M Karri RR Roy S, editors. Environmental and Health Management of Novel Coronavirus Disease (COVID-19). Cambridge, MA: Academic Press (2021). p. 305–24.

[5] Price JC Forrest JS. Chapter 12 - Airport Emergency Planning, Part III. In:Price JC Forrest JS, editors. Practical Airport Operations, Safety, and Emergency Management. Oxford, United Kingdom: Butterworth-Heinemann (2016). p. 489–556.

[6] Ceylan Z Ocak E Uçar Y Karakus K Cetinkaya T. Chapter 12—An overview of food safety and COVID-19 infection: nanotechnology and cold plasma applications, immune-boosting suggestions, hygienic precautions. In:Hadi Dehghani M Karri RR Roy S, editors. Environmental and Health Management of Novel Coronavirus Disease (COVID-19). Cambridge, MA: Academic Press (2021). p. 325–344

[7] Chidume CG Oko-Otu CN Aro GC. State Fragility and Covid-19 pandemic: Implications on the political economy of Nigeria. Soc Sci Human Open. (2021) 3:100127. 10.1016/j.ssaho.2021.100127 34173511 PMC7890347

[8] Ejeromedoghene O Tesi JN Uyanga VA Adebayo AO Nwosisi MC Tesi GO. Food security and safety concerns in animal production and public health issues in Africa: a perspective of COVID-19 pandemic era. Ethics Med Public Health. (2020) 15:100600. 10.1016/j.jemep.2020.100600 33015275 PMC7523516

[9] Dardaque Mucinhato RM da Thimoteo Cunha D Fernandes Barros SC Zanin LM Auad LI Cezimbra Weis GC. Behavioral predictors of household food-safety practices during the COVID-19 pandemic: extending the theory of planned behavior. Food Control. (2021) 108719. 10.1016/j.foodcont.2021.108719 34961805 PMC8695225

[10] Soon JM Vanany I Abdul Wahab IR Hamdan RH Jamaludin MH. Food safety and evaluation of intention to practice safe eating out measures during COVID-19: Cross sectional study in Indonesia and Malaysia. Food Control. (2021) 125:107920. 10.1016/j.foodcont.2021.107920 35668872 PMC9159731

[11] Duda-Chodak A Lukasiewicz M Zieć G Florkiewicz A Filipiak-Florkiewicz A. Covid-19 pandemic and food: Present knowledge, risks, consumers fears and safety. Trends Food Sci Technol. (2020) 105:145–60. 10.1016/j.tifs.2020.08.020 32921922 PMC7480472

[12] Han S Roy PK Hossain MI Byun KH Choi C Ha SD. COVID-19 pandemic crisis and food safety: Implications and inactivation strategies. Trends Food Sci Technol. (2021) 109:25–36. 10.1016/j.tifs.2021.01.004 33456205 PMC7794057

[13] Byrd K Her E Fan A Almanza B Liu Y Leitch S. Restaurants and COVID-19: what are consumers' risk perceptions about restaurant food and its packaging during the pandemic? Int J Hosp Manage. (2021) 94:102821. 10.1016/j.ijhm.2020.102821 34866742 PMC8631525

[14] Dedeoglu BB Bogan E. The motivations of visiting upscale restaurants during the COVID-19 pandemic: the role of risk perception and trust in government. Int J Hosp Manage. (2021) 95:102905. 10.1016/j.ijhm.2021.102905 PMC9756826 36540678

[15] Thomas MS Feng Y. Consumer risk perception and trusted sources of food safety information during the COVID-19 pandemic. Food Control. (2021) 130:108279. 10.1016/j.foodcont.2021.108279 PMC9759357 36568483

[16] Coppola DP. Chapter 6—response. In:Coppola DP, editor. Introduction to International Disaster Management (Fourth Edition). Oxford, United Kingdom: Butterworth-Heinemann (2020). p. 393–470, e326.

[17] Limon MR. Food safety practices of food handlers at home engaged in online food businesses during COVID-19 pandemic in the Philippines. Curr Res Food Sci. (2021) 4:63–73. 10.1016/j.crfs.2021.01.001 33665620 PMC7903060

[18] de Freitas RSG Stedefeldt E. COVID-19 pandemic underlines the need to build resilience in commercial restaurants' food safety. Food Res Int. (2020) 136:109472. 10.1016/j.foodres.2020.109472 32846557 PMC7319922

[19] Brooks C Parr L Smith JM Buchanan D Snioch D Hebishy E. A review of food fraud and food authenticity across the food supply chain, with an examination of the impact of the COVID-19 pandemic and Brexit on food industry. Food Control. (2021) 130:108171. 10.1016/j.foodcont.2021.108171

[20] Burgos D Ivanov D. Food retail supply chain resilience and the COVID-19 pandemic: a digital twin-based impact analysis and improvement directions. Transp Res Part E Logist Transp Rev. (2021) 152:102412. 10.1016/j.tre.2021.102412 34934397 PMC8677600

[21] Chitrakar B Zhang M Bhandari B. Improvement strategies of food supply chain through novel food processing technologies during COVID-19 pandemic. Food Control. (2021) 125:108010. 10.1016/j.foodcont.2021.108010 33679006 PMC7914018

[22] Marti L Puertas R García-Álvarez-Coque JM. The effects on European importers' food safety controls in the time of COVID-19. Food Control. (2021) 125:107952. 10.1016/j.foodcont.2021.107952 33584020 PMC7869612

[23] Halabowski D Rzymski P. Taking a lesson from the COVID-19 pandemic: Preventing the future outbreaks of viral zoonoses through a multi-faceted approach. Sci Total Environ. (2021) 757:143723. 10.1016/j.scitotenv.2020.143723 33213901 PMC7666614

[24] Angouria-Tsorochidou E Thomsen M. Modelling the quality of organic fertilizers from anaerobic digestion—Comparison of two collection systems. J Clean Prod. (2021) 304:127081. 10.1016/j.jclepro.2021.127081

[25] Rodrigues D Teixeira R Shockley J. Inspection agency monitoring of food safety in an emerging economy: a multilevel analysis of Brazil's beef production industry. Int J Prod Econ. (2019) 214:1–16. 10.1016/j.ijpe.2019.03.024

[26] Hong W Mao J Wu L Pu X. Public cognition of the application of blockchain in food safety management—Data from China's Zhihu platform. J Clean Prod. (2021) 303:127044. 10.1016/j.jclepro.2021.127044

[27] Kaur K Randhawa G. Exploring the influence of supportive supervisors on organisational citizenship behaviours: Linking theory to practice. IIMB Manage Rev. (2021) 33:156–65. 10.1016/j.iimb.2021.03.012

[28] Wang Q An D Wen L Shi Y Meng Y Lu W. Food hygiene supervision during a major conference in Beijing: descriptive analysis of impact on risk factors. Food Control. (2012) 28:279–85. 10.1016/j.foodcont.2012.05.053

[29] Yang XT Qian JP Li J Ji ZT Fan Bl Xing B. A real-time agro-food authentication and supervision system on a novel code for improving traceability credibility. Food Control. (2016) 66:17–26. 10.1016/j.foodcont.2016.01.032

[30] Bowles A. Enforcement authority perspective on the food manufacturing sector (UK EHO). In:Swainson M, editor. Swainson's Handbook of Technical and Quality Management for the Food Manufacturing Sector. Sawston, United Kingdom: Woodhead Publishing (2019). p. 385–410.

[31] He J. A review of Chinese fish trade involving the development and limitations of food safety strategy. Ocean Coast Manage. (2015) 116:150–61. 10.1016/j.ocecoaman.2015.07.017

[32] Ryan JM. Chapter 1 - Background: Understanding common and assignable causes, laws, and costs. In:Ryan JM, editor. Validating Preventive Food Safety and Quality Controls. Cambridge, MA: Academic Press (2017). p. 1–29.

[33] Falguera V Aliguer N Falguera M. An integrated approach to current trends in food consumption: moving toward functional and organic products? Food Control. (2012) 26:274–81. 10.1016/j.foodcont.2012.01.051

[34] Tulchinsky TH Varavikova EA. Chapter 3—Measuring, monitoring, and evaluating the health of a population. In:Tulchinsky TH Varavikova EA, editors. The New Public Health (Third Edition). Cambridge, MA: Academic Press (2014). p. 91–147.

[35] Dong YY Liu JH Wang S Chen Q-l Guo TY Zhang LY. Emerging frontier technologies for food safety analysis and risk assessment. J Integr Agric. (2015) 14:2231–42. 10.1016/S2095-3119(15)61123-6

[36] Gray J Hu Y Wilson A Chandry PS Tinoco MB Jordan KN. 1.07—The role of genomics in food quality and safety management: possibilities and limitations. In:Cifuentes A, editor. Comprehensive Foodomics. Elsevier (2021). p. 127–37.

[37] Krishnaswami A Beavers C Dorsch MP Dodson JA Masterson Creber R Kitsiou S. Gerotechnology for older adults with cardiovascular diseases: JACC state-of-the-art review. J Am Coll Cardiol. (2020) 76:2650–70. 10.1016/j.jacc.2020.09.606 33243384 PMC10436190

[38] Sepahvand M Abdali-Mohammadi F. A novel multi-lead ECG personal recognition based on signals functional and structural dependencies using time-frequency representation and evolutionary morphological CNN. Biomed Signal Process Control. (2021) 68:102766. 10.1016/j.bspc.2021.102766

[39] Diehlmann F Lüttenberg M Verdonck L Wiens M Zienau A Schultmann F. Public-private collaborations in emergency logistics: a framework based on logistical and game-theoretical concepts. Saf Sci. (2021) 141:105301. 10.1016/j.ssci.2021.105301

[40] Lakitan B Hidayat D Herlinda S. Scientific productivity and the collaboration intensity of Indonesian universities and public RandD institutions: are there dependencies on collaborative RandD with foreign institutions? Technol Soc. (2012) 34:227–38. 10.1016/j.techsoc.2012.06.001

[41] Gauri V. Redressing grievances and complaints regarding basic service delivery. World Dev. (2013) 41:109–19. 10.1016/j.worlddev.2012.05.027

[42] Linde L Sjödin D Parida V Wincent J. Dynamic capabilities for ecosystem orchestration A capability-based framework for smart city innovation initiatives. Technol Forecast Soc Change. (2021) 166:120614. 10.1016/j.techfore.2021.120614

[43] Savaglio C Ganzha M Paprzycki M Bădică C Ivanović M Fortino G. Agent-based Internet of Things: State-of-the-art and research challenges. Future Gener Comput Syst. (2020) 102:1038–53. 10.1016/j.future.2019.09.016

[44] Bickley SJ Torgler B. A systematic approach to public health—Novel application of the human factors analysis and classification system to public health and COVID-19. Saf Sci. (2021) 140:105312. 10.1016/j.ssci.2021.105312 33897105 PMC8053242

[45] Henrique de Moura E Bruno Rocha e Cruz T De Genaro Chiroli DM. A framework proposal to integrate humanitarian logistics practices, disaster management and disaster mutual assistance: a Brazilian case. Saf Sci. (2020) 132:104965. 10.1016/j.ssci.2020.104965

[46] Marques CM Moniz S de Sousa JP Barbosa-Povoa AP Reklaitis G. Decision-support challenges in the chemical-pharmaceutical industry: Findings and future research directions. Comput Chem Eng. (2020) 134:106672. 10.1016/j.compchemeng.2019.106672

[47] Miranda BV Monteiro GFA Rodrigues VP. Circular agri-food systems: a governance perspective for the analysis of sustainable agri-food value chains. Technol Forecast Soc Change. (2021) 170:120878. 10.1016/j.techfore.2021.120878

[48] Nyarugwe SP Linnemann AR Ren Y Bakker EJ Kussaga JB Watson D. An intercontinental analysis of food safety culture in view of food safety governance and national values. Food Control. (2020) 111:107075. 10.1016/j.foodcont.2019.107075

[49] Martindale L. From land consolidation and food safety to taobao villages and alternative food networks: four components of China's dynamic agri-rural innovation system. J Rural Stud. (2021) 82:404–16. 10.1016/j.jrurstud.2021.01.012

[50] Thomas MJ Lal V Baby AK Rabeeh Vp M James A Raj AK. Can technological advancements help to alleviate COVID-19 pandemic? A review. J Biomed Inform. (2021) 117:103787. 10.1016/j.jbi.2021.103787 33862231 PMC8056973

[51] Hsu B-X Chen Y-M Chen L-A. Corporate social responsibility and value added in the supply chain: Model and mechanism. Technol Forecast Soc Change. (2022) 174:121302. 10.1016/j.techfore.2021.121302

[52] Luger M Hofer KM Floh A. Support for corporate social responsibility among generation Y consumers in advanced versus emerging markets. Int Bus Rev. (2021) 101903. 10.1016/j.ibusrev.2021.101903

[53] Hassauer C Roosen J. Toward a conceptual framework for food safety criteria: analyzing evidence practices using the case of plant protection products. Saf Sci. (2020) 127:104683. 10.1016/j.ssci.2020.104683

[54] Sun D Liu Y Grant J Long Y Wang X Xie C. Impact of food safety regulations on agricultural trade: Evidence from China's import refusal data. Food Policy. (2021) 105:102185. 10.1016/j.foodpol.2021.102185

[55] Trienekens J Zuurbier P. Quality and safety standards in the food industry, developments and challenges. Int J Prod Econ. (2008) 113:107–22. 10.1016/j.ijpe.2007.02.050

[56] Bentia DC. Accountability beyond measurement. The role of meetings in shaping governance instruments and governance outcomes in food systems through the lens of the Donau Soja organisation. J Rural Stud. (2021) 88:50–9. 10.1016/j.jrurstud.2021.09.026

[57] Vara-Sánchez I Gallar-Hernández D García-García L Morán Alonso N Moragues-Faus A. The co-production of urban food policies: exploring the emergence of new governance spaces in three Spanish cities. Food Policy. (2021) 103:102120. 10.1016/j.foodpol.2021.102120

[58] Abu Hatab A Cavinato MER Lindemer A Lagerkvist C-. J. (2019). Urban sprawl, food security and agricultural systems in developing countries: a systematic review of the literature. Cities 94:129–42. 10.1016/j.cities.2019.06.001

[59] Ogunniyi AI Mavrotas G Olagunju KO Fadare O Adedoyin R. Governance quality, remittances and their implications for food and nutrition security in Sub-Saharan Africa. World Dev. (2020) 127:104752. 10.1016/j.worlddev.2019.104752

[60] Lee J Huang Y-H Dainoff MJ He Y. Where to focus? Insights from safety personnel and external safety consultants on lessons learned about safety climate interventions—A qualitative approach. J Saf Res. (2021) 79:51–67. 10.1016/j.jsr.2021.08.005 34848020

[61] Pahl-Wostl C. The role of governance modes and meta-governance in the transformation towards sustainable water governance. Environ Sci Policy. (2019) 91:6–16. 10.1016/j.envsci.2018.10.008

[62] Lin CF. Chapter 15 - The Emergence and Influence of Transnational Private Regulation of Food Safety^**^A previous version of this chapter was published by the Food and Drug Law Institute in Volume 69, Issue 2, of the Food and Drug Law Journal. The author thanks the Food and Drug Law Journal for the permission to adapt this Chapter from the publication. In:Halabi SF, editor. Food and Drug Regulation in an Era of Globalized Markets. Cambridge, MA: Academic Press (2015). p. 183–203.

[63] Hennessy DA Marsh TL. Chapter 79 - Economics of animal health and livestock disease. In:Barrett CB Just DR, editors. Handbook of Agricultural Economics. Amsterdam, Netherlands: Elsevier (2021). p. 4233–330.

[64] Friswell R Williamson A. Management of heavy truck driver queuing and waiting for loading and unloading at road transport customers' depots. Saf Sci. (2019) 120:194–205. 10.1016/j.ssci.2019.06.039

[65] Williams KG. Chapter 55 - Laws governing the practice of pharmacy. In:Adejare A, editor. Remington (Twenty-third Edition). Cambridge, MA: Academic Press (2021). p. 929–65

[66] Krzysztof F. Chapter 10—The openness and cooperation in the food sector. In:Galanakis CM, editor. Innovation Strategies in the Food Industry (Second Edition). Cambridge, MA: Academic Press (2022). p. 157–69.

[67] Liu H Wang J Wu Y Zhang L. The follow-up evaluation of “General Hygienic Regulation for Food Production” in China. Food Control. (2018) 93:70–5. 10.1016/j.foodcont.2018.05.033

[68] Zhao X Wang P Pal R. The effects of agro-food supply chain integration on product quality and financial performance: Evidence from Chinese agro-food processing business. Int J Prod Econ. (2021) 231:107832. 10.1016/j.ijpe.2020.107832

[69] Chaudhary S Dhir A Ferraris A Bertoldi B. Trust and reputation in family businesses: a systematic literature review of past achievements and future promises. J Bus Res. (2021) 137:143–61. 10.1016/j.jbusres.2021.07.052

[70] Gross HP Ingerfurth S Willems J. Employees as reputation advocates: Dimensions of employee job satisfaction explaining employees' recommendation intention. J Bus Res. (2021) 134:405–13. 10.1016/j.jbusres.2021.05.021

[71] ElBagoury M Tolba MM Nasser HA Jabbar A Elagouz AM Aktham Y. The find of COVID-19 vaccine: Challenges and opportunities. J Infect Public Health. (2021) 14:389–416. 10.1016/j.jiph.2020.12.025 33647555 PMC7773313

[72] Newell-McGloughlin M Burke J. Biotechnology crop adoption: potential and challenges of genetically improved crops. In:Van Alfen NK, editor. Encyclopedia of Agriculture and Food Systems. Cambridge, MA: Academic Press (2014). p. 69–93.

[73] Ashley JM. Chapter five—prevention of future food insecurity. In:Ashley JM, editor. Food Security in the Developing World. Cambridge, MA: Academic Press (2016). p. 81–140.

[74] Gordon A Gordon D. Chapter 3 - Food safety and quality systems implementation along value chains. In:Gordon A, editor. Food Safety and Quality Systems in Developing Countries. Cambridge, MA: Academic Press (2020). p. 81–124.

[75] Chen K Wang XX Song HY. Food safety regulatory systems in Europe and China: a study of how co-regulation can improve regulatory effectiveness. J Integr Agric. (2015) 14:2203–17. 10.1016/S2095-3119(15)61113-3

[76] Li Y Qi R Liu H. Designing Independent Regulatory System of Food Safety in China. Agricult Agricult Sci Procedia. (2010) 1:288–95. 10.1016/j.aaspro.2010.09.036

[77] Sundqvist-Andberg H Åkerman M. Sustainability governance and contested plastic food packaging—An integrative review. J Clean Prod. (2021) 306:127111. 10.1016/j.jclepro.2021.127111

[78] Unnevehr L Hoffmann V. Food safety management and regulation: International experiences and lessons for China. J Integr Agric. (2015) 14:2218–30. 10.1016/S2095-3119(15)61112-126029897

[79] Kokkinakis E Kokkinaki A Kyriakidis G Markaki A Fragkiadakis GA. HACCP implementation in local food industry: a survey in Crete, Greece. Procedia Food Sci. (2011) 1:1079–83. 10.1016/j.profoo.2011.09.161

[80] Varzakas TH Tsigarida ET Apostolopoulos C Kalogridou-Vassiliadou D Jukes DJ. The role of the Hellenic Food Safety Authority in Greece—Implementation strategies. Food Control. (2006) 17:957–65. 10.1016/j.foodcont.2005.07.00236423648

[81] Charnes A Cooper WW Rhodes E. Measuring the efficiency of decision making units. Eur J Oper Res. (1978) 2:429–44. 10.1016/0377-2217(78)90138-8

[82] Farrell MJ. The measurement of productive efficiency. J Royal Statist Soc Ser A. (1957) 120:253–81. 10.2307/2343100

[83] Førsund F Kittelsen S Krivonozhko V. Farrell revisited-Visualizing properties of DEA production frontiers. J Oper Res Soc. (2009) 60:1535–45. 10.1057/jors.2008.185

[84] Vanpoucke E Vereecke A Wetzels M. Developing supplier integration capabilities for sustainable competitive advantage: A dynamic capabilities approach. J Operat Manag. (2014) 32:446–61. 10.1016/j.jom.2014.09.004

[85] Nakku VB Agbola FW Miles MP Mahmood A. The interrelationship between SME government support programs, entrepreneurial orientation, and performance: a developing economy perspective. J Small Bus Manage. (2020) 58:2–31. 10.1080/00472778.2019.1659671

[86] Christensen T Lægreid P Rykkja LH. Organizing for crisis management: building governance capacity and legitimacy. Public Admin Rev. (2016) 76:887–97. 10.1111/puar.12558

[87] Novickytė L Droždz J. Measuring the efficiency in the lithuanian banking sector: The DEA application. Int J Finan Stud. (2018) 6:37. 10.3390/ijfs6020037

